# Anti-Carbamylated LL37 Antibodies Promote Pathogenic Bone Resorption in Rheumatoid Arthritis

**DOI:** 10.3389/fimmu.2021.715997

**Published:** 2021-09-14

**Authors:** Liam J. O’Neil, Christopher B. Oliveira, Donavon Sandoval-Heglund, Ana Barrera-Vargas, Javier Merayo-Chalico, Eduardo Aguirre-Aguilar, Mariana J. Kaplan, Carmelo Carmona-Rivera

**Affiliations:** ^1^Systemic Autoimmunity Branch, National Institute of Arthritis and Musculoskeletal and Skin Diseases, National Institutes of Health, Bethesda, MD, United States; ^2^Department of Immunology and Rheumatology, Instituto Nacional de Ciencias Medicas y de la Nutricion, Salvador Zubiran, Mexico City, Mexico

**Keywords:** LL-37, carbamylation, neutrophils, rheumatoid arthritis, NETs

## Abstract

**Objective:**

Antibodies against carbamylated proteins (anti-CarP) are associated with poor prognosis and the development of bone erosions in rheumatoid arthritis (RA). RA neutrophils externalize modified autoantigens through the formation of neutrophil extracellular traps (NETs). Increased levels of the cathelicidin LL37 have been documented in the synovium of RA patients, but the cellular source remains unclear. We sought to determine if post-translational modifications of LL37, specifically carbamylation, occur during NET formation, enhance this protein’s autoantigenicity, and contribute to drive bone erosion in the synovial joint.

**Methods:**

ELISA and Western blot analyses were used to identify carbamylated LL37 (carLL37) in biological samples. Anti-carLL37 antibodies were measured in the serum of HLA-DRB1*04:01 transgenic mice and in human RA synovial fluid.

**Results:**

Elevated levels of carLL37 were found in plasma and synovial fluid from RA patients, compared to healthy controls. RA NETs release carLL37 and fibroblast-like synoviocytes (FLS) internalized NET-bound carLL37 and loaded it into their MHCII compartment. HLA-DRB1*04:01 transgenic mice immunized with FLS containing NETs developed autoantibodies against carLL37. Anti-carLL37 antibodies were present in RA sera and synovial fluid and they correlated with radiologic bone erosion scores of the hands and feet in RA patients. CarLL37-IgG immune complexes enhanced the ability of monocytes to differentiate into osteoclasts and potentiated osteoclast-mediated extracellular matrix resorption.

**Conclusions:**

NETs are a source of carLL37 leading to induction of anti-carbamylated autoantibody responses. Furthermore, carLL37-IgG immune complexes may be implicated in the bone damage characteristic of RA. These results support that dysregulated NET formation has pathogenic roles in RA.

## Introduction

Rheumatoid arthritis (RA) is an autoimmune disease characterized by inflammation of the joint, cartilage damage and bone erosion (1). Lack of appropriate control of RA symptomatology is associated with joint destruction, disability and increased mortality. One of the hallmarks of RA is the presence of autoantibodies to post-translationally modified proteins (2), particularly directed against citrulline. More recently, antibodies against a similar but structurally distinct modification, homocitrulline (carbamylation), termed anti-CarP have been described in several cohorts of RA patients (3–5). The presence of anti-carbamylated protein autoantibodies (anti-CarP) is associated with enhanced radiographic bone erosion (3); however, the pathogenic mechanisms underlying this observation are not well understood.

Neutrophils are highly abundant in the synovial fluid of RA patients (6) and we previously reported that RA neutrophils display an enhanced capacity to form neutrophil extracellular traps (NETs) and that these structures are a source of both citrullinated and carbamylated autoantigens (7, 8). NETs carrying modified autoantigens can be internalized by fibroblast-like synoviocytes (FLS), endowing them with antigen presenting cell-capabilities and induction of anti-citrulline pathogenic adaptive immunity (9). Carbamylation is a non-enzymatic posttranslational modification (PTM) of a positively charged lysine residue, which yields neutral charged homocitrulline. Carbamylation can also occur at sites of inflammation, possibly due to cyanate formation during neutrophil oxidative burst (10, 11). The relative contribution of PTM’s in NET-associated proteins remains unknown, and how these modified proteins drive aspects of disease pathogenesis requires further exploration.

LL37 is an antimicrobial peptide that is externalized during NET formation and is elevated in the synovium of RA patients (12, 13). LL37 PTMs can impair its antimicrobial capacity (11), while autoantibodies against LL37 have been implicated in the pathogenesis of autoimmune diseases such as systemic lupus erythematosus (SLE) (12, 14–16). Furthermore, carbamylation of LL37 and antibodies against carLL37 have been reported in psoriatic arthritis patients (17) but their role in disease pathogenesis is unclear.

Here, we sought to investigate the role of carbamylated LL37 (carLL37) in the pathogenesis of RA. Specifically, we hypothesized that NETs are a source of carLL37 and that this autoantigen may mediate a pathogenic immune response and be critical for the development of erosive joint disease.

## Materials and Methods

### Human Specimens and Cells

Patients recruited in this study fulfilled the 2010 American College of Rheumatology criteria for RA (18). Healthy controls were recruited by advertisement. All individuals gave written informed consent and enrolled in a protocol approved by the Instituto Nacional de Ciencias Medicas y de la Nutrición Salvador Zubirán (INCMNSZ, Ref 1243). A complete clinical examination was performed by a rheumatologist, which included documentation of the Disease Activity Score (DAS-28) (19). Hand and foot RA radiographs were scored using the Simple Erosion Narrowing Score (SENS) (20, 21). The rheumatologist who scored the radiographs was blinded to the patients’ clinical data. Patient characteristics can be found in **Table S1**. Peripheral blood (PB) was obtained by venipuncture and collected in EDTA-containing tubes. PB was fractionated *via* Ficoll-Paque Plus (GE Healthcare) gradient. Neutrophils were isolated by dextran sedimentation and hypotonic salt solution as previously described (7). Healthy control PB CD14^+^ were purified by positive selection. Briefly, PBMCs were incubated with CD14 beads (Miltenyi Biotech) in MACS buffer and isolated according to manufacturer’s instructions by positive selection. Synovial fluid was collected from a separate Canadian cohort (22) (Ethics Board approval number HS14453) of RA patients. Samples were collected by routine joint aspiration, aliquoted, labelled by diagnosis and stored at -20 until further use. For the purposes of this study, samples were classified as either RA or non-RA (5.8% Psoriatic Arthritis, 5.8% Polymyalgia Rheumatica, 5.8% Reactive Arthritis, 11.8% Connective tissue disease, and 70.6% Osteoarthritis).

### Quantification of Serum Carbamylated LL37 and NET Complexes

A 96-well plate was coated with rabbit polyclonal carbamylated-Lysine antibody (Cell bioLabs) at 1:400 in PBS overnight at 4°C. Wells were washed and blocked with 1% BSA at room temperature for 1 hour. Diluted serum (1:100) was added to the wells in 1% BSA blocking buffer and incubated overnight at 4°C. The wells were washed three times and incubated with mouse monoclonal anti-LL37 (EMD Millipore) at 1:100 in blocking buffer. After washing three times, goat anti-mouse conjugated HRP antibody (Bio-Rad) was added to the wells in blocking buffer at 1: 10,000. Wells were washed five times, followed by the addition of TMB substrate (Sigma Aldrich) and stop solution (Sigma Aldrich). The absorbance was measured at 450 nm and values were calculated as an OD index. The OD index is calculated by normalizing all OD to the control mean (OD index = OD value/control OD mean). Assays were performed in duplicate.

For NET complexes, a similar procedure was followed using a 96-well plate that was coated with either carbamylated-Lysine antibody (Cell bioLabs) or rabbit anti-citrullinated histone 3 (Abcam) in PBS overnight at 4°C. Mouse monoclonal anti-dsDNA (EMD Millipore) was used as primary antibody diluted (1:100) in blocking buffer followed by incubation with goat anti-mouse conjugated HRP antibody (Bio-Rad) at 1: 10,000 dilution. OD index for ELISA is calculated using the following formula: OD index value = OD value/control OD mean.

### Effect of Immune Complexes on Osteoclast Formation

A 96-well plate was coated with 200 ng of carbamylated LL37 in PBS overnight. LL37 immune complexes were generated by adding 100 ug of total IgG isolated from RA serum using the Melon kit (Thermo Fisher). After two hours incubation, wells were washed with PBS. CD14+ cells were isolated as described above and incubated in the presence of 50ng/mL of monocyte colony stimulating factor (M-CSF) for three days. Pre-osteoclasts were seeded in the with carbamylated LL37-coated wells in the presence or absence of RA IgGs. Cells were cultured with M-CSF and RANKL (100 ng/mL). After four days, the plate was washed and a TRAP staining kit (Kamiya Biomedical company) was used to detect TRAP-positive cells, indicative of OCs. Multinucleated TRAP positive cells were quantified and plotted.

### Immunofluorescence of NET Treated Fibroblast-Like Synoviocytes

Methods for these experiments were adopted from our group’s previous work (9). Briefly, FLS obtained from RA patients were cultured on coverslips and treated with either RA NETs or vehicle. For plasma membrane detection, cells were incubated with membrane-dye (Biotium) for 30 min at 37 C. Cell were washed and fixed with 4% paraformaldehyde 12h at 4°C. For intracellular detection, cells were permeabilized with 0.2% triton for 10 min at room temperature. Coverslips were blocked with porcine gelatin (Sigma) for 30 minutes, then incubated for 1 hour with the primary antibody [anti-LL37 (Abcam) or anti-MHCII (Abcam)] at 37°C. After washing, coverslips were incubated for 30 minutes with secondary antibodies, then counterstained with Hoechst 1:1000. After further washing, coverslips were mounted on glass slides using Prolong-gold (Invitrogen). Images were acquired on a Zeiss LSM 780 confocal microscope.

### Immunization of HLA-DRB4*04:01 Transgenic Mouse Model

HLA-DRB4*04:01 breeding pairs were a kind gift from Dr. Chella David (Mayo Clinic) and were housed and bred at the NIH animal facility. Animal studies were conducted according to the guidelines established by the NIAMS Laboratory Animal Care and Use section and following approved protocol (A016-05-26). Mouse FLS (100 000 per well) were cultured in the presence or absence of 50 μg of spontaneously generated human RA NETs for three days prior to injection. FLS were washed with PBS, detached with Trypsin, washed, and resuspended in PBS. FLS with and without NETs were injected into the hindleg synovial space using a 27-gauge needle. This procedure was performed every 7 days for a total of 7 weeks (7 injections). Serum was collected for analysis of autoantibodies.

### OC Resorption Assay

CD14+ cells were isolated from PBMC from healthy control using MACs columns. Cells were incubated in the presence of 50ng/mL of M-CSF for three days, followed by incubation with 100ng/mL of RANKL for 7 days. OCs were detached using non-enzymatic dissociation solution (Sigma). Carbamylated LL37 was bound to a calcium phosphate coated plate overnight at 4°C, washed, and incubated with 100 ug of purified RA IgG (Melon IgG spin purification kit) for 60 minutes at room temperature. Plate was washed extensively with PBS to remove non-specific binding. Equal numbers of OCs were seeded in a calcium-phosphate coated plate (KT651 Kamiya) in the presence or absence of 200 ng of carbamylated LL37 or carbamylated LL37-IgG. OCs were cultured in the presence of RANKL (100 ng/mL) for 3 days. Cells were removed, and the plate was washed and scanned in a Keyence microscope. Images were analyzed using ImageJ.

### Western Blotting

Neutrophils from healthy volunteers or RA patients were resuspended in RPMI and seeded in 24-well plates in the presence or absence of 100 ng/mL of PMA (Sigma) or 2.5 uM of calcium Ionophore (Sigma) for 4 hours at 37°C. NETs were harvested with 10 U/mL of micrococcal nuclease (ThermoFisher, Waltham, MD) for 10 min at 37°C. NETs were collected and cleared of debris by centrifugation. NETs were quantified using a BCA protein assay (Pierce) and equal amounts of NET protein were resolved in a 4-12% gradient Bis-Tris gel (Invitrogen), transferred onto a nitrocellulose membrane and blocked with 10% BSA for 30 min at room temperature. After overnight incubation with primary antibodies, membranes were washed three times with PBS-Tween (PBS-T) and incubated with secondary antibody coupled to IRDye 800CW. Membranes were developed using Li-COR Odyssey Clx scanner (Li-COR).

### Statistical Analysis

All analyses were performed using GraphPad Prism Version 8.1.1 (La Jolla, CA) unless otherwise stated. Mann-Whitney U test was used for non-parametric continuous comparisons between 2 groups. Continuous associations were assessed by linear regression using base R. Code is available upon request. All analyses were considered statistically significant at p < 0.05.

## Results

### Carbamylated LL37 Is Elevated in RA Serum, Synovial Fluid, and NETs

**Figure 1:**
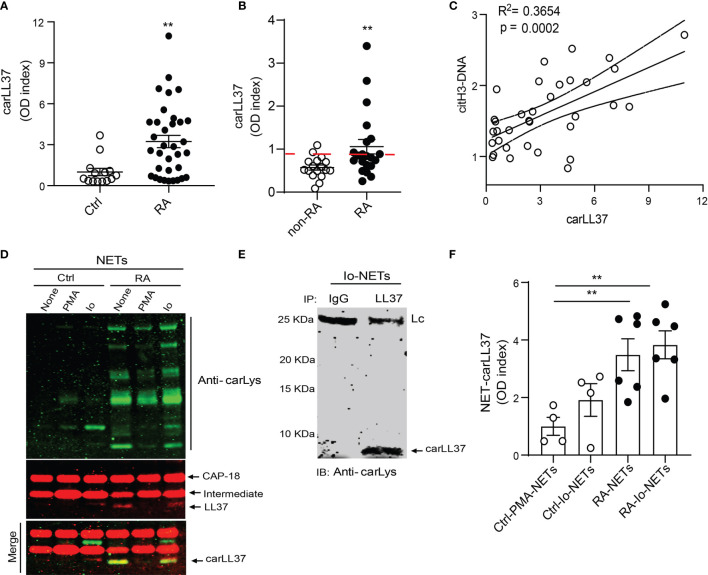
Carbamylated LL37 is externalized in NETs and is elevated in RA serum and synovial fluid. Carbamylated LL37 was measured in **(A)** serum from healthy controls (n = 25) and RA patients (n = 34) and **(B)** synovial fluid (SF) from non-RA (n = 10) and RA (n = 17). Red dashed line = 2 standard deviations above mean for non-RA patients. **(C)** Citrullinated histone H3-DNA complexes correlate with levels of carbamylated LL37 in serum in RA (n = 34). **(D)** Western blot analysis of carbamylated proteins in NETs isolated from control or RA neutrophils in the presence or absence of PMA and ionophore (Io). Red: CAP18, intermediate and LL37, green: carbamylated lysine, yellow: carbamylated LL37. Bottom panel (merge) red bands are full length CAP18 and intermediate, yellow is carbamylated LL37 **(E)** Ionophore generated NETs were immunoprecipitated against LL37 and developed against carbamylation (carLys) by western blot. Lc is the light chain of the antibody used to immunoprecipitate. **(F)** Carbamylated LL37 was measured by ELISA in NETs isolated from control neutrophils stimulated with PMA or calcium ionophore (Io) or RA neutrophils in the presence or absence or calcium ionophore (Io). Results represent the mean +/- SEM unless otherwise stated. Mann-Whitney U test was used for between group comparisons. **p < 0.01.

We have previously shown that carbamylated histones are found in NETs (8), and these antigens potentiate RA bone erosion. However, little is known about carbamylated LL37 and its relevance in RA pathogenesis, despite it being highly expressed in the RA synovium and known component of NETs (13). Thus, we investigated whether carbamylated LL37 can also be detected in RA. By ELISA, we detected significant elevation of carLL37 in RA serum when compared to serum from healthy volunteers (**Figure 1A**). Similarly, levels of carLL37 were significantly elevated in synovial fluid from RA patients when compared to non-RA synovial fluid (**Figure 1B**). Since LL37 can be externalized during NET formation (23), we explored the possibility that NETs also contain the carbamylated form of LL37. We found a significant positive correlation between serum levels of citrullinated histone 3/DNA complexes, a previously validated surrogate marker of NETs, and serum levels of carbamylated LL37 (**Figure 1C**). These results suggested that NETs are a source of carLL37 in RA (**Figure 1C**).

Healthy control and RA neutrophils were isolated and stimulated with phorbol 12-myristate 13-acetate (PMA) or calcium ionophore A23187 for 4 hours to induce NET formation. Western blot analysis showed that spontaneously generated RA NETs (without PMA or ionophore *in vitro* addition) contained elevated amounts of carbamylated proteins, when compared to healthy control NETs induced in the presence of either PMA or calcium ionophore (**Figure 1D**). Moreover, Western blot analysis showed that LL37 was carbamylated in RA NETs (**Figure 1D**). Anti-carbamylated antibody did not cross-react with citrullinated residues, suggesting it is specific for the recognition of carbamylated proteins (**Figure S1**). Interestingly, supernatant collected from healthy control neutrophils also contained carbamylated LL37, suggesting that carLL37 may be released during degranulation and NET formation (**Figure S2**). To further demonstrate that LL37 was carbamylated, immunoprecipitation was performed. Western blot analysis of immunoprecipitated LL37 demonstrated carbamylation of the protein (**Figure 1E**). Quantification of carLL37 in NETs by ELISA showed significant elevation in spontaneously generated RA NETs when compared to healthy control NETs generated with either PMA or calcium ionophore (**Figure 1F**). These data suggest that RA neutrophils extrude carLL37 during NET formation.

### FLS Internalize carLL37 and Promote Anti Car-Autoantibody Responses

**Figure 2:**
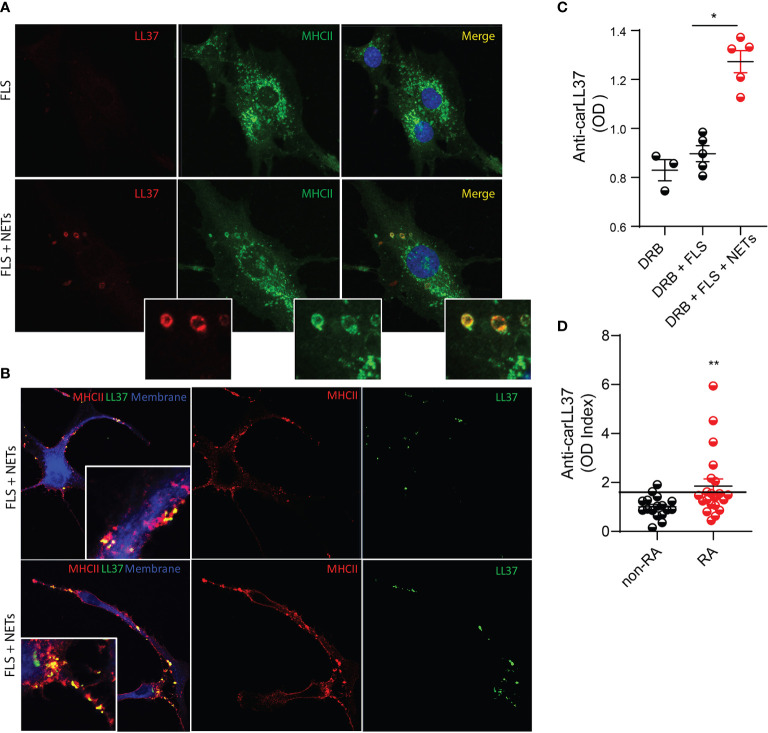
FLSs internalize carbamylated LL37 and induce antigen-specific autoantibody responses. NETs containing carbamylated LL37 were incubated with RA FLS for 24 hours. **(A)** Intracellular co-localization of carbamylated LL37 and MHCII was assessed in RA FLS. Red, LL37; green, MHCII; blue, DNA. **(B)** Co-localization of MHCII and LL37 was assessed in the plasma membrane of un permeabilized RA FLS after 48h incubation with NETs. Green, LL37; red, MHCII; blue, plasma membrane. Results are representative of three independent experiments. Original magnification is 400X. **(C)** Serum samples from HLA-DRB1*04:01 transgenic mice immunized with FLS alone or FLS that internalized RA-NETs were analyzed for the presence of autoantibodies against carbamylated LL37. Results are the mean +/- SEM of n=3-5 per group. One-way ANOVA- Dunn’s multiple comparisons test was used. *p < 0.05. **(D)** Autoantibodies against carbamylated LL37 were measured in non-RA (n = 9) and RA (n = 15) synovial fluid. Black line = 2 standard deviations above mean for non-RA patients. Results are the mean +/- SEM. Mann-Whitney U test was used. **p < 0.01.

Carbamylated NET proteins contribute to the pool of RA autoantigens (8), but how these modified proteins stimulate adaptive immune responses remains unclear. We previously reported that FLS can acquire antigen presenting cell capabilities by internalizing NETs and presenting NET-derived citrullinated antigens to antigen-specific CD4+ T cells (9). We then investigated whether FLS can also internalize NET-bound carLL37, and whether this internalization can also induce antigen-specific adaptive immune responses. Immunofluorescence confocal microscopy analysis demonstrated that LL37 can be internalized by RA FLS and that it colocalizes with the MHCII compartment (**Figure 2A**). This suggests that carLL37 can be loaded onto MHCII compartment intracellularly. In addition, colocalization of LL37 and MHCII was also detected in the plasma membrane of nonpermeabilized FLS (**Figure 2B**), suggesting that the LL37/MHCII complex traffics to the plasma membrane.

To test whether FLS that have internalized NETs can induce specific adaptive immune responses *in vivo*, we used the humanized HLA-DRB4*04:01 transgenic mouse model (24). We previously showed that FLS isolated from these mice can internalize RA NETs (9). We isolated FLS from HLA-DRB4*04:01 transgenic mice and incubated them with spontaneously generated RA NETs for 24h. A total of 100,000 FLS, with or without internalized NETs, were injected into one knee joint of each HLA-DRB4*04:01 mouse. After seven rounds of injections, antibodies against carLL37 were measured. Significantly higher levels of anti-carLL37 antibodies were detected in the sera of mice that received intra-articular injection of FLS loaded with NETs, when compared with animals that received FLS alone (**Figure 2C**). To recapitulate these findings in the human synovium, we quantified autoantibodies against carLL37 in synovial fluid from RA and non-RA subjects. Significantly higher levels of anti-carLL37 autoantibodies were detected in RA synovial fluid when compared to non-RA SF. These results suggest that RA patients also develop antigen-specific adaptive immune responses to carbamylated LL37 (**Figure 2D**).

### Anti-Carbamylated LL37 Antibodies Correlate With Radiographic Bone Erosions in RA

**Figure 3:**
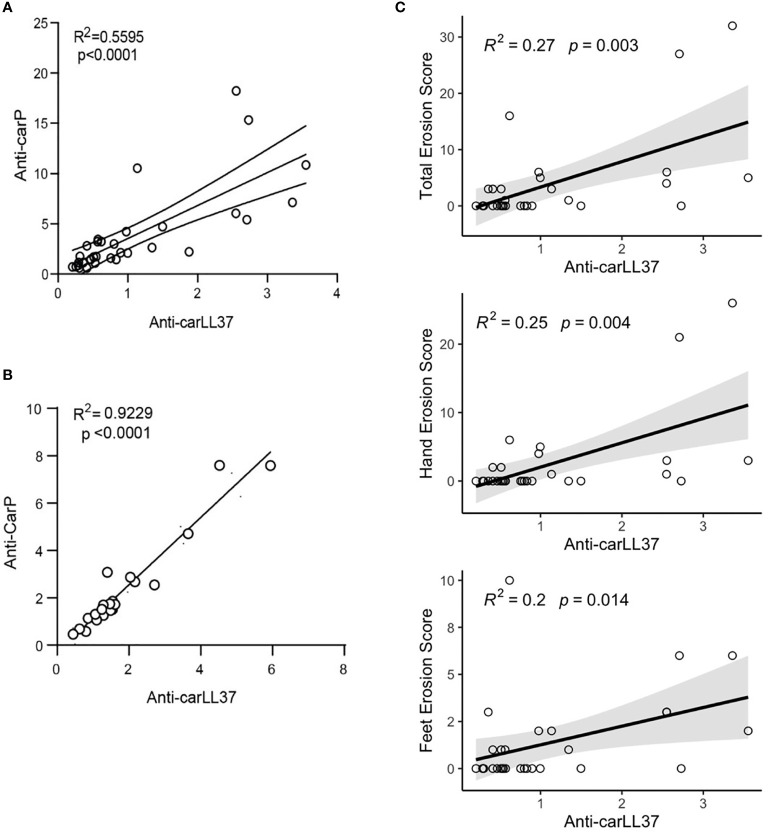
Anti-carLL37 antibodies correlate with radiographic erosion scores in RA. Correlation of anti-CarP antibodies with antibodies against carbamylated LL37 in **(A)** serum and **(B)** synovial fluid of RA patients (n = 15). **(C)** Correlation of total (top), hand (middle) or feet (bottom) radiographic erosions scores and antibodies against carbamylated LL37.

To assess the clinical significance of anti-carLL37 responses, we first assessed the correlation between anti-CarP and anti-carLL37 responses in RA. A positive correlation was found between the anti-CarP and anti-carLL37 levels in RA serum (**Figure 3A**) and synovial fluid (**Figure 3B**). To investigate the clinical relevance of presence and levels of anti-carLL37 antibodies in RA, we performed supervised correlations between anti-carLL37, carLL37 protein and several clinical parameters (**Table S2**). Although smoking status has been associated with risk of developing antibodies against carbamylated proteins (25), we did not find a significant correlation between smoking and the presence of anti-carLL37 Ab (**Table S2**). A positive correlation was found between serum levels of anti-carLL37 Abs and the presence of periarticular hand (p=0.004) and foot (p = 0.01) bone erosions and radiographic erosion score (p = 0.003, **Figure 3C**). Interestingly, the strength of this association exceeded that of anti-carbamylated Histone Ab (R^2^ 0.27 *vs* 0.23 respectively, **Table S2**), which have been previously shown to associate with erosive disease (8). These results suggest that anti-carLL37 autoantibodies may be implicated in the development of bone erosions in RA, and suggest that anti-carLL37 may be considered as a clinically useful biomarker for risk of erosive RA.

### Carbamylated LL37-IgG Immune Complexes Enhance OC Formation and Activity

**Figure 4:**
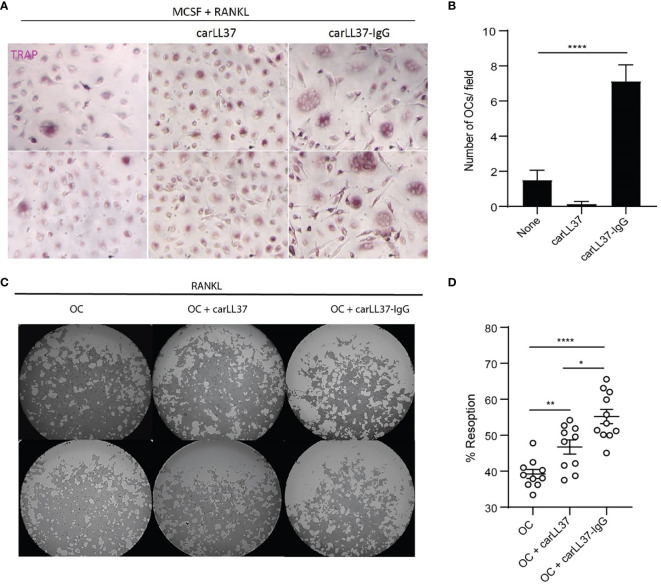
CarLL37-IgG immune complexes enhance osteoclast formation and function. **(A)** Representative pictures of M-CSF/RANKL-mediated osteoclastogenesis in the presence or absence of anti-carbamylated LL37 IgG immune complexes. Original magnification is 400X. **(B)** Quantification of TRAP-positive multinucleated cells. Results are the mean +/- SEM of 4-5 independent experiments. Kruskal-Wallis test was used. ****p < 0.0001. **(C)** Representative pictures of resorption pit (white areas) assay of equal number of M-CSF/RANKL generated osteoclasts (OC) in the presence or absence of anti-carbamylated LL37-IgG immune complexes in a calcium-phosphate plate. **(D)** Percentage of eroded surface using ImageJ. Results are the mean +/- SEM of 10 independent experiments. Mann-Whitney U-test was used. *p < 0.05, **p < 0.01, ****p < 0.0001.

Given the relationship between anti-carLL37 Abs and radiographic bone erosions, we sought to determine if these antibodies directly impacted OC formation and bone resorption capabilities. We have previously shown that carbamylated-histone-IgG complexes potentiate OC formation and activity (8). Whether other carbamylated IgG complexes also increase OC function is not known. Healthy control PB CD14+ monocytes were incubated with M-CSF/RANKL in the presence or absence of carLL37-IgG immune complexes. A significant increase in multinucleated TRAP-positive cells was found in the cells exposed to anti-carLL37-IgG immune complexes, when compared to carLL37 alone or in cells only treated with M-CSF/RANKL (**Figures 4A, B**). These results suggest that the presence of carLL37-IgG immune complexes accelerates OC formation. Next, equal number of OCs (generated by MCSF/RANKL) were plated on a calcium-phosphate plate in the presence or absence of carLL37 or carLL37-IgG immune complexes. RANKL was added to all conditions to activate OCs. CarLL37-IgG immune complexes significantly enhanced OC resorptive activity from 40% to 65%, when compared to OC without immune complexes (**Figures 4C, D**). Of note, carLL37 alone was also able to significantly increase OC resorption (**Figure 4D**). These results suggest that anti-carLL37-IgG immune complexes potentiate OC formation and activity.

## Discussion

Increasing evidence supports the notion that neutrophils are key players in the generation of modified autoantigens in the RA synovium (7, 9). While various post-translationally modified antigens are present in NETs, much of the research has focused on citrullinated autoantigens. Recent evidence suggests that other post-translational modifications may also play an important role in RA pathogenesis. Carbamylation is a modification that is structurally similar to citrulline, is also generated in proinflammatory environments, and potentially links smoking and/or diet to RA development. Our prior work suggests that carbamylated histones, amongst other carbamylated autoantigens, are generated during NET formation. We found that LL37 is also carbamylated in spontaneously generated RA NETs. FLS that internalize NET fragments containing carLL37 were loaded onto the cells MHCII compartment, trafficking to the FLS plasma membrane which might be presented to CD4+T cells as we have shown previously (9). This process leads to the generation of anti-carLL37 antibodies in the synovium of RA patients, which are correlated with radiographic bone erosion in an RA cohort. Furthermore, carLL37-IgG immune complexes potentiate OC formation and their ability to resorb bone, providing a mechanistic link between specific autoantibody responses and clinical outcomes.

Large number of activated neutrophils are found in the synovial fluid of RA patients during active and in early phases of the disease (6, 26) and they associate with the development of classic RA manifestations such as morning stiffness (27). LL37 is highly expressed in inflamed RA synovial joints as well as in pristane-induced arthritis rat models (1, 16). Antibodies to carbamylated proteins has a significant association with enhanced radiographic bone erosions in RA (3, 5). We now provide evidence that anti-carLL37 antibodies strongly associates with anti-CarP responses in serum and synovial fluid and, most importantly, correlates with bone radiographic scores. A possible mechanism for this association is that carLL37-IgG immune complexes, similar to other antigen-IgG complexes in RA, may enhance osteoclast differentiation and function in the RA synovium. This data builds on our previous observations and provides evidence that antibody responses to a variety of PTM’s may potentiate mechanisms of bone erosions. Importantly, our previous work that elucidates the mechanism by which carbamylated histones enhances OC activity, strengthens the observation that innate immune proteins derived from NETs can breach immune tolerance and potentiate bone resorption, playing a dual role in RA. Further studies interrogating the specificity of these antigen-Ig interactions are required to better understand their precise role in RA pathogenesis. This finding opens the possibility that other posttranslational modifications may contribute to joint damage in this disease.

HLA-DRB1*04:01 transgenic mice immunized with FLS loaded with RA NETs developed antibodies against carLL37. This supports the notion that genetic susceptibility and environmental factors are crucial in the interplay of autoantibody generation against post-translationally modified neoantigens. FLS-T-cell interactions may be critical for the loss of immune tolerance, and it remains to be determined if other cell-cell interactions are important for these events to occur (28). Of note, the presence of anti-carLL37 antibodies has also been documented in psoriatic arthritis, suggesting that additional genetic polymorphisms besides the shared epitope may be implicated in these responses (29). Furthermore, the bone damage phenotype in psoriatic arthritis is different from the one in RA and further understanding how anti-CarP responses modulate bone damage in other diseases need to be further investigated. Finally, the cross-reactive nature of anti-modified citrullinated antibodies is an important consideration when interpreting these results, however it remains unclear if autoantibodies that target carbamylated residues also cross-react, and to what degree, in anti-carP positive patients (30).

The results from this study further support the rationale for testing whether inhibitors of dysregulated NET formation (31), targeting neutrophil hyperactivity, and/or preventing specific cell-cell interactions in the synovium in future clinical trials in RA.

## Data Availability Statement

The raw data supporting the conclusions of this article will be made available by the authors, without undue reservation.

## Ethics Statement

The studies involving human participants were reviewed and approved by Instituto Nacional de Ciencias Medicas y de la Nutrición Salvador Zubirán (INCMNSZ, Ref 1243). The patients/participants provided their written informed consent to participate in this study. The animal study was reviewed and approved by NIAMS Laboratory Animal Care and Use section and following approved protocol (A016-05-26).

## Author Contributions

LO’N, CO, DS-H, and CC-R performed the experiments. LO’N, AB-V, JM-C, EA-A, and CC-R analyzed the data and performed statistical analyses. AB-V, JM-C, and EA-A provided specimens and clinical information. LO’N and CC-R were involved in overall design and/or manuscript preparation. LO’N, MK, and CC-R drafted the manuscript. All authors contributed to the article and approved the submitted version.

## Funding

Supported by the Intramural Research Program, NIAMS/NIH, ZIA AR041199.

## Conflict of Interest

The authors declare that the research was conducted in the absence of any commercial or financial relationships that could be construed as a potential conflict of interest.

## Publisher’s Note

All claims expressed in this article are solely those of the authors and do not necessarily represent those of their affiliated organizations, or those of the publisher, the editors and the reviewers. Any product that may be evaluated in this article, or claim that may be made by its manufacturer, is not guaranteed or endorsed by the publisher.
