# Supplementary material for: Anti-Carbamylated LL37 Antibodies Promote Pathogenic Bone Resorption in Rheumatoid Arthritis
Source: Front Immunol. 2021 Sep 14;12:715997. doi: 10.3389/fimmu.2021.715997 (PMC8477029; doi:10.3389/fimmu.2021.715997)
Supplement: Supplementary file 1 [file DataSheet_1.docx]

**Anti-carbamylated LL37 antibodies promote pathogenic bone resorption in Rheumatoid Arthritis.**

Liam J. O’Neil^1^, Christopher B. Oliveira^1^, Donavon Sandoval-Heglund^1^, Ana Barrera-Vargas^2^, Javier Merayo-Chalico^2^, Eduardo Aguirre-Aguilar^2^, Mariana J. Kaplan^1*^, Carmelo Carmona-Rivera^1*^

**Supplementary Tables and Figures**

| RA cohort (n = 34) | |
| --- | --- |
| Age | 47.5 (15.3) |
| Female | 85.3% |
| BMI | 26.3 (5.9) |
| Disease Duration (years) | 5 (9.3) |
| RF + | 91.1% |
| ACPA + | 91.1% |
| DAS28-CRP | 2.0 (2.3) |
| Smoker | 35.3% |
| Methotrexate | 67.7% |
| Prednisone > 10 mg daily | 0.0% |
| Biologic | 3.2% |
| Radiographic Erosions | 50.0% |
| SENS range | 0 to 37 |

Table S1: **Demographics and clinical characteristics of Rheumatoid Arthritis cohort.** Continuous variables are listed as median with interquartile range (IQR). Dichotomized variables are listed by % affirmative. DAS28: Disease Activity Score. Biologic is considered active use at the time of visit. RF: Rheumatoid Factor. ACPA: Anti-citrullinated protein antibody. Radiographic erosion score defined using Simple erosion narrowing score (SENS).

|  | Age | Sex | BMI | Smoker | Disease Duration | ACPA | RF | DAS28 | Hand erosion | Feet erosion | Total erosion | Anti-carH4 | Anti-carLL37 |
| --- | --- | --- | --- | --- | --- | --- | --- | --- | --- | --- | --- | --- | --- |
| Age | 1.00 | 0.12 | 0.13 | -0.03 | 0.17 | -0.03 | -0.20 | 0.09 | 0.33 | 0.33 | 0.34 | 0.01 | 0.11 |
| Sex | 0.12 | 1.00 | -0.32 | -0.24 | -0.18 | -0.30 | -0.31 | 0.33 | -0.24 | -0.19 | -0.24 | 0.03 | -0.04 |
| BMI | 0.13 | -0.32 | 1.00 | -0.14 | -0.04 | 0.06 | -0.17 | 0.11 | 0.09 | 0.18 | 0.11 | 0.14 | 0.18 |
| Smoker | -0.03 | -0.24 | -0.14 | 1.00 | 0.25 | 0.20 | -0.12 | -0.14 | 0.11 | 0.00 | 0.09 | -0.19 | -0.15 |
| Disease Duration | 0.17 | -0.18 | -0.04 | 0.25 | 1.00 | 0.35 | 0.16 | 0.21 | 0.87 | 0.72 | **0.86** | 0.58 | 0.69 |
| ACPA | -0.03 | -0.30 | 0.06 | 0.20 | 0.35 | 1.00 | -0.12 | -0.21 | 0.41 | 0.32 | **0.40** | 0.04 | 0.11 |
| RF | -0.20 | -0.31 | -0.17 | -0.12 | 0.16 | -0.12 | 1.00 | -0.13 | 0.04 | 0.07 | 0.04 | 0.37 | 0.32 |
| DAS28 | 0.09 | 0.33 | 0.11 | -0.14 | 0.21 | -0.21 | -0.13 | 1.00 | 0.07 | 0.13 | 0.08 | 0.30 | 0.25 |
| Hand erosion | 0.33 | -0.24 | 0.09 | 0.11 | 0.87 | 0.41 | 0.04 | 0.07 | 1.00 | 0.83 | **0.99** | 0.43 | 0.59 |
| Feet erosion | 0.33 | -0.19 | 0.18 | 0.00 | 0.72 | 0.32 | 0.07 | 0.13 | 0.83 | 1.00 | **0.89** | 0.51 | 0.67 |
| Total erosion | 0.34 | -0.24 | 0.11 | 0.09 | 0.86 | 0.40 | 0.04 | 0.08 | 0.99 | 0.89 | 1.00 | 0.46 | 0.63 |
| Anti-carH4 | 0.01 | 0.03 | 0.14 | -0.19 | 0.58 | 0.04 | 0.37 | 0.30 | 0.43 | 0.51 | **0.46** | 1.00 | 0.96 |
| Anti-carLL37 | 0.11 | -0.04 | 0.18 | -0.15 | 0.69 | 0.11 | 0.32 | 0.25 | 0.59 | 0.67 | **0.63** | 0.96 | 1.00 |

Table S2: Correlation matrix displaying clinical variables and autoantibodies against citrulline (ACPA), carbamylated Histone 4 (Anti-carH4) and carbamylated LL37 (Anti-carLL37). R values are displayed. Bolded parameters show possible biomarkers for erosive RA based on radiographic scoring. BMI: body mass index, DAS28: Disease activity score.


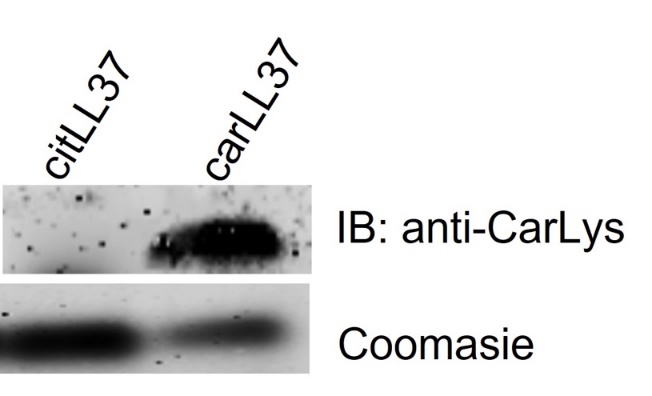


Figure S1. **Antibody specificity.** To assess whether antibodies distinguish between citrullination and carbamylation (homocitrulline), recombinant LL37 was incubated in the presence of PAD2 or presence of cyanate to generate citrullinate and carbamylated form of LL37 respectively. Western blot analysis shows that anti-carLys antibody recognizes the carbamylated form of LL37, but not the citrullinated form. Coomasie blue was used as loading control to detect total recombinant LL37 loaded per lane.


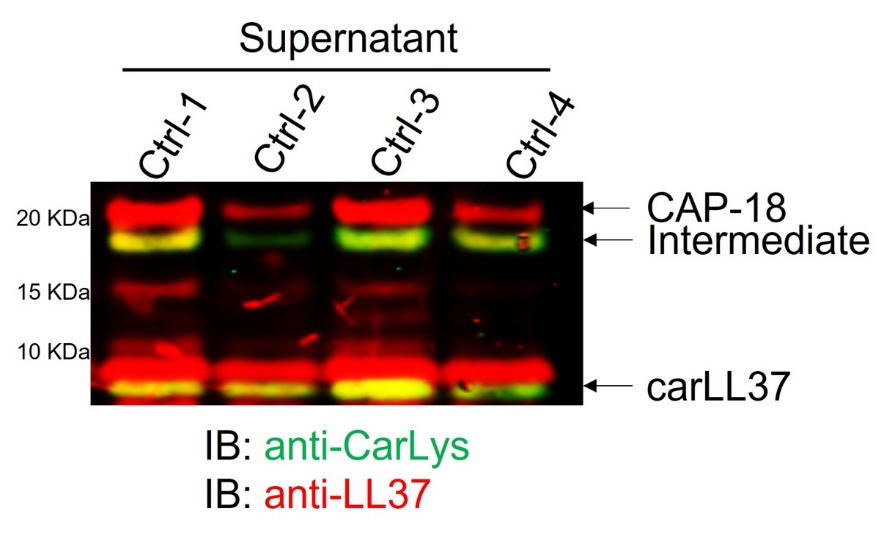


Figure S2: Western blot of supernatant derived from neutrophils stimulated with Ionophore for 4 hours. Red: LL37, Green: carbamylated-lysine, yellow: carbamylated LL37. CAP-18: full length precursor cathelicidin.
